# Reduced Graphene Oxide-Coated Si Nanowires for Highly Sensitive and Selective Detection of Indoor Formaldehyde

**DOI:** 10.1186/s11671-019-2921-2

**Published:** 2019-03-14

**Authors:** Longfei Song, Linqu Luo, Yan Xi, Jianjun Song, Ying Wang, Liping Yang, Anqi Wang, Yunfa Chen, Ning Han, Fengyun Wang

**Affiliations:** 1 College of Physics and State Key Laboratory of Bio-Fibers and Eco-Textiles, Qingdao University, Qingdao, 266071 China; 2 State Key Laboratory of Multiphase Complex Systems, Institute of Process Engineering, Chinese Academy of Sciences, Beijing, 100190 China; 3 Center for Excellence in Regional Atmospheric Environment, Institute of Urban Environment, Chinese Academy of Sciences, Xiamen, 361021 China; 4 Key Laboratory of Microelectronic Devices and Integrated Technology, Institute of Microelectronics, Chinese Academy of Sciences, Beijing, 100029 China

**Keywords:** Si nanowires, Reduced graphene oxide, Sensitivity, Selectivity, Formaldehyde

## Abstract

Although significant developments have been made in the low-concentration formaldehyde monitoring in indoor air by using gas sensors, they still suffer from insufficient performance for achieving ppb-level detection. In this work, <100> oriented Si nanowires (SiNWs) with high specific surface area were prepared via metal-assisted chemical etching method (MACE), and then were uniformly coated with graphene oxide (GO) followed by the subsequent reductive process in H_2_/Ar atmosphere at 800 °C to obtain reduced graphene oxide (RGO). The RGO coating (RGO@n-SiNWs) obviously enhances SiNWs sensitivity to low-concentration formaldehyde, benefiting from the increased specific surface area, the sensitization effect of RGO, and the formation of p-n junction between SiNWs and RGO. Specifically, RGO@n-SiNWs exhibits a high response of 6.4 to 10 ppm formaldehyde at 300 °C, which is about 2.6 times higher than that of pristine SiNWs (~ 2.5). Furthermore, the RGO@n-SiNWs show a high response of 2.4 to 0.1 ppm formaldehyde which is the largest permissive concentration in indoor air, a low detection limit of 35 ppb obtained by non-linear fitting, and fast response/recovery times of 30 and 10 s. In the meanwhile, the sensor also shows high selectivity over other typical interfering gases such as ethanol, acetone, ammonia, methanol, xylene, and toluene, and shows a high stability over a measurement period of 6 days. These results enable the highly sensitive, selective, and stable detection of low-concentration formaldehyde to guarantee safety of indoor environment.

**Electronic supplementary material:**

The online version of this article (10.1186/s11671-019-2921-2) contains supplementary material, which is available to authorized users.

## Introduction

Nowadays, as one of the toxic volatile organic compounds (VOCs) in newly built house environment, formaldehyde (HCHO) is seriously threatening human health [1–12], which is considered to be one of the major sources of sick building syndrome (SBS) [13, 14], and a carcinogen by International Agency for Research on Cancer (IAIC) [2]. Therefore, several standards have been set up to avoid the risk induced by the pollution of indoor air. In the literatures, the upper limit of formaldehyde concentration established by the National Institute for Occupational Safety and Health (NIOSH) is 0.1 ppm in the living room and 1 ppm in industrial production workshop [2]. Meanwhile, the World Health Organization (WHO) also established a safe standard of 0.08 ppm averaged over 30 min for long-term exposure in formaldehyde vapor [15]. Therefore, the successful detection of low-concentration HCHO makes a great stride to ensure the safety of living environment.

Although many schemes have been developed for detecting low-concentration HCHO, including liquid chromatograph (LC) [16, 17], spectroscopy [9], etc., these techniques have limitations for portable use and real-time monitoring due to their bulky sizes and complicated analysis processes [18]. Currently, gas sensors based on the semiconductor nanostructures (e.g., In_2_O_3_ [19, 20], Cr_2_O_3_ [20], SnO_2_ [21–23]) are extensively employed in the detection of low-concentration HCHO, owing to their high sensitivity, fast response, and excellent chemical stability [2, 10, 19, 24–32]. These sensors based on semiconductor nanostructures offer significant advantages compared with LC and spectroscopy, such as easy miniaturization for portable use, low cost, and in-situ detection. However, their responses to HCHO need to be further improved at the ppb level though they are good at ppm level. For example, Chen et al. reported Ga-doped In_2_O_3_ nanofiber sensors which showed a high response (defined as *R*_a_/*R*_g_, where the *R*_a_ and *R*_g_ are the resistances of the sensor in air and in HCHO) of 52.4 to 100 ppm HCHO, while < 1.5 to 0.1 ppm, which needs to be enhanced to meet the response requirement of practical utilization limitation of *R*_a_/*R*_g_ = 2 [19]. Therefore, it is an urgent affair to find an efficient route to enhance the sensitivity for reaching the safe detection limitation. Silicon nanowires (Si NWs) have been selected as one of semiconductor materials to be used in chemical sensors. For example, biosensors based on chemically modified Si NW field effect transistors have been reported and demonstrated a superior sensitivity and selectivity to proteins [33]. However, this sensor fabrication needs a high cost and complicated process as the sensitivity has to be improved by the filed effect.

Recently, the incorporation of graphene with nanostructured semiconductor gas sensors becomes a promising approach to improve the sensitivity, due to its high specific surface area and exceptional sensitivity to gases [34]. Compared with the sensitization effect of conventional noble metals (e.g., Pt, Pd, and Au nanoparticles) [35–37], this strategy can not only possess the merits of low cost and high efficiency but also enlarge the surface area and improve the electron transport. For example, reduced graphene oxide (RGO)-SnO_2_ [18], RGO-Cu_2_O [38], graphene-SnO_2_ [39] have demonstrated excellent enhancement of gas sensitivity. However, many reports put the semiconductor nanostructures on the surface of RGO or graphene to form simple contact, of which the efficient contact area is too restricted to achieve the maximization of sensitivity. Therefore, it is significant to search an efficient and feasible strategy to realize core-shell structures based on RGO and semiconductor.

In this work, highly sensitive and selective detection of low-concentration HCHO was achieved by a core-shell structure of RGO-coated silicon nanowires (SiNWs), with increased specific surface area twice as large as SiNWs. Specifically, the response of reduced graphene oxide-coated n-type silicon nanowires (RGO@n-SiNWs) increases about 2.6× toward 10 ppm HCHO (~ 6.4) than that of pristine SiNWs (~ 2.5) at the best operation temperature of 300 °C, which is attributed to the excellent sensitization effect of RGO. The as-fabricated sensors can reach a superior application detection limitation of as low as 35 ppb, and the response/recovery times are as fast as 30/10 s. Besides the improved sensitivity, the selectivity is high over typical interfering gases (e.g., ethanol, acetone, ammonia, methanol, xylene, and toluene) and the stability is good in a period of 6 days. All of the results made a significant stride toward using reduced graphene oxide-coated silicon nanowires (RGO@SiNWs) for the low concentration HCHO detection in indoor environment.

## Materials and Methods

### Fabrications of SiNWs Arrays

n (100) and p (100) Silicon wafers (0.005–0.02 Ωcm and 0.001–0.005 Ωcm) were employed as starting wafers (3.0 cm × 3.0 cm). Before the etching process, the Si wafers were cleaned in acetone for 10 min, ethanol for 10 min, and deionized (DI) water for 10 min in turn. The cleaned starting wafers were immersed in oxidant solution containing H_2_SO_4_ (97%, Sigma-Aldrich) and H_2_O_2_ (35%, GR 30 wt.% in H_2_O, Aldrich) in a volume ratio of 3:1 for 30 min to remove the organic contaminants on the surface. After the cleaning step, the samples were then immersed into 5% HF solution for 8 min at room temperature to dissolve the thin oxide layer formed on the surface and thus the fresh Si surfaces were H-terminated. Next, the cleaned Si wafers were immediately transferred into an Ag coating solution containing 0.005 M AgNO_3_ (99.99%, Aladdin) and 4.8 M HF (Aladdin, GR 40%), which was slowly stirred for 1 min at room temperature (~25 ^o^C). After a uniform layer of Ag nanoparticles (AgNPs) was deposited on the surfaces, the AgNPs-coated wafers were washed with deionized water to remove the extra Ag^+^ ions. Then, the wafers were etched in the etching solution (H_2_O_2_ = 0 .4 M and HF = 4 .8 M) for 30 min at room temperature in the dark. Finally, the samples were dipped in the aqueous solution of HNO_3_ (70%, Sigma-Aldrich) to dissolve the Ag catalyst, and then rinsed with deionized water for several times to remove residual layer. The fabricated SiNWs were slowly scraped by a sharp blade.

### SiNWs Functionalized with RGO

The graphene oxide (GO) dispersion was synthesized by the modified Hummer’s method [40], and then was ultrasonically dispersed in 60 mL DI water for 3 h to prepare the GO solution (30 mg). In a typical synthesis, the obtained SiNWs (0.2 g) were firstly dispersed in the mixture of DI water (10 mL) and ethanol (30 mL), then ethylenediamine (400 μL) was dropwise added. After the ultrasonic treatment for 20 min, 20 mL GO solution was added to the above solution and kept vigorous stirring. Subsequently, the product was collected by centrifugation and washed with ethanol for several times, then dried at 60 °C to obtain GO@SiNWs. Finally, the GO@SiNWs was reduced in H_2_/Ar atmosphere at 800 °C (2 °C min^−1^) to obtain RGO@SiNWs.

### Characterization of SiNWs and RGO@SiNWs

The morphology of SiNWs and RGO@SiNWs was observed by scanning electron microscopy (SEM, JSM-7001F+INCA X-MAX) and transmission electron microscopy (TEM, JEM-2100F). Besides, the crystal structure was studied by X-ray diffraction (XRD, X’Pert PRO MPD). Additionally, in order to analyze the surface area and pore size distribution, nitrogen absorption-desorption isotherm was performed on a specific area and a pore-size analyzer (SSA-7300, BUILDER) by the Brunauer–Emmett–Teller (BET) method and Barett–Joyner–Halenda (BJH) model, respectively. For the confirmation of the existence of RGO, Raman spectrum was performed by a Raman spectrometer (Thermo Scientific DXR2). Besides, the elemental analyses were performed by X-ray photoelectron spectroscopy (XPS, ESCALAB 250, Al Kα radiation).

### Devices Fabrication and Measurement

As-prepared RGO@SiNWs (~ 5 mg) was mixed with ethanol (~ 100 μL), and dispersed uniformly by ultrasonic. The dispersed solution was coated onto a ceramic plate with Pt wires (i.e., heater and measurer), and aged under a voltage of 5 V for 3 days in air. Finally, the prepared devices were measured in a gas sensor analyzer (Winsen WS-30A, China). Formaldehyde was produced by the evaporation of formaldehyde solution (40 wt%) at heating holder in chamber. Ethanol, acetone, ammonia, methanol, xylene, and toluene were produced by pure liquid ethanol, acetone, ammonia, methanol, xylene, and toluene respectively. Response is defined as *R*_a_/*R*_g_, where *R*_a_ and *R*_g_ are the resistances of the sensor in pure air and in formaldehyde gases. Response/Recovery times are defined as the time needed to change to 90% of the total response.

## Results and Discussions

**Fig. 1:**
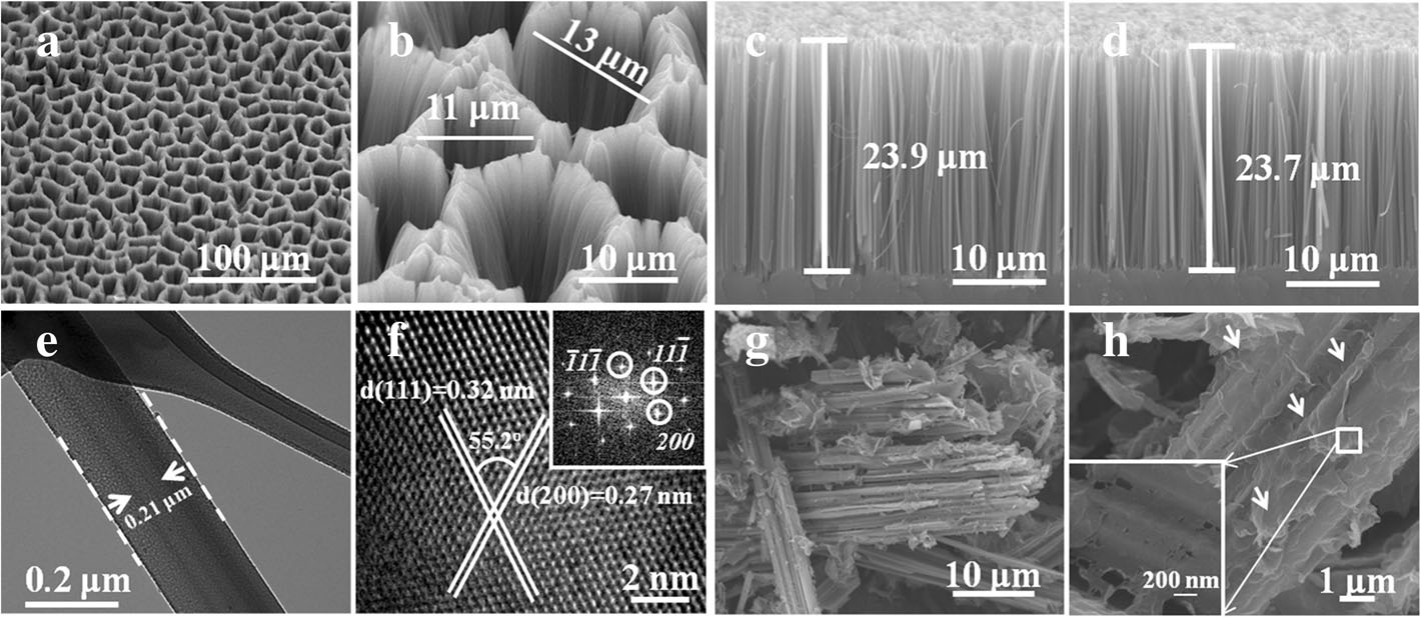
**a** Top view, **b** zoomed-in top view, and **c** cross-sectional SEM images of n-SiNWs. **d** Cross-sectional SEM image of p-SiNWs. **e** TEM image of n-SiNWs. **f** HRTEM image of n-SiNWs together with the corresponding FFT. **g** SEM image of RGO@n-SiNWs with HF treatment. **h** Zoomed-in SEM image of RGO@n-SiNWs with HF treatment

In order to study the morphologies and microstructures, SEM and TEM were performed as shown in Fig. 1. Figure 1a displays the large-scale top view SEM image of as-prepared SiNWs, showing the uniform surface and congregated bundles of SiNWs due to the electrostatic attraction among SiNWs [41, 42]. There are full of large pores with the size of 2~15 μm on the surface as observed in zoomed-in SEM in Fig. 1b. As depicted in the cross-section SEM images of n- and p-SiNWs in Fig. 1c, d, the etched NWs are all perpendicular to the smooth substrate, proving the same <100> orientation as the starting wafer. In addition, the similar length of ~ 24 μm, diameter of 100~300 nm, and density of approximately 10^10^ cm^−2^ [41] were demonstrated distinctly, which indicates no difference between <100> oriented n- and p-SiNWs. The scraped n- and p- SiNWs are observed in Additional file 1: Figure S1a and b, which reflect no morphology change after scripting. In order to further confirm the diameter and orientation, TEM images of single n- and p-SiNWs display the diameter of 210 nm (Fig. 1e) and 200 nm (Additional file 1: Figure S2a), respectively. Figure 1f and Additional file 1: Figure S2b are high-resolution TEM (HRTEM) images together with the Fast Fourier Transfer (FFT), measuring the single crystalline structure and the <100> crystal orientation with the (200) spacing of 0.27 nm. The underlying mechanism of SiNWs fabrications using metal-assisted chemical etching (MACE) method is a series of simple redox reactions with the help of Ag catalysts, which can be described briefly by Eq. 1 and Eq. 2.

Reaction at metal (i.e., Ag particles):

1 $$ {\mathrm{H}}_2{\mathrm{O}}_2\kern0.5em +\kern0.5em 2{\mathrm{H}}^{+}\kern0.5em \to \kern0.5em 2{\mathrm{H}}_2\mathrm{O}\kern0.5em +\kern0.5em 2{\mathrm{h}}^{+}\kern0.5em \mathrm{and}\kern0.5em 2{\mathrm{H}}^{+}\kern0.5em +\kern0.5em 2{\mathrm{e}}^{\hbox{-}}\kern0.5em \to \kern0.5em 2{\mathrm{H}}_2 $$

Reaction at Si substrate:

2 $$ \mathrm{Si}\kern0.5em +\kern0.5em 4{\mathrm{h}}^{+}\kern0.5em +\kern0.5em 4\mathrm{HF}\kern0.5em \to \kern0.5em {\mathrm{SiF}}_4\kern0.5em +\kern0.5em 4{\mathrm{H}}^{+}\kern0.5em \mathrm{and}\kern0.5em {\mathrm{SiF}}_4\kern0.5em +\kern0.5em 2\mathrm{HF}\kern0.5em \to \kern0.5em {\mathrm{H}}_2{\mathrm{SiF}}_6 $$

Throughout this process, Ag nanoparticles directly seize electrons from Si because of the higher electronegativity of Ag compared with Si, creating a hole-rich region around the Ag nanoparticles. Then, H_2_O_2_ is reduced by Ag nanoparticles and Si is oxidized to be SiO_2_, which is dissolved quickly by HF solution [43].

Next, the as-etched SiNWs were functionalized by RGO. Figure 1g is the SEM image of RGO@n-SiNWs and Fig. 1h is the zoomed SEM images of RGO@n-SiNWs, which proved that RGO was compactly and uniformly wrapped on the surface of NWs. There would be a formation of p-n junction between RGO and SiNWs, which is important for the enhancement of sensors sensitivity discussed in the following sections.

**Fig. 2:**
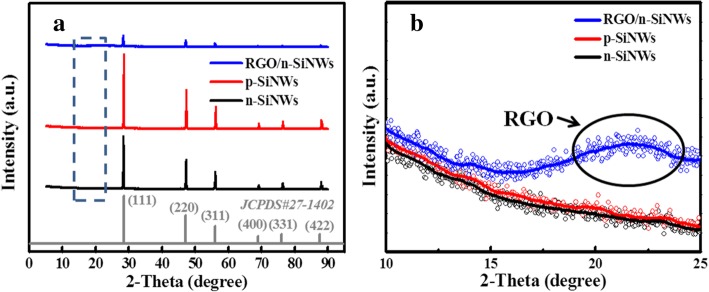
**a** XRD patterns of n-/p-SiNWs and RGO@n-SiNWs. **b** Zoomed-in XRD patterns from 10 to 25 degree

To shed light on the components and crystallinity, X-ray diffraction (XRD) patterns are performed as shown in Fig. 2a. For n- and p-SiNWs, the main peaks locate at 28.4°, 47.3°, 56.1°, 69.1°, 76.4°, and 88.0°, corresponding to (111), (200), (400), (331), and (422) planes of cubic silicon structure (JCPDS No. 27-1402), respectively. There was no impurity peak observed, indicating the purity of the samples. The XRD pattern of RGO@n-SiNWs also exhibits the same peaks. Obviously, it is found that the peak intensities of RGO@n-SiNWs declined distinctly, which was attributed to the existence of outer amorphous RGO. In order to confirm that GO was entirely reduced to RGO, the zoomed-in XRD spectra from 10° to 25° were shown in Fig. 2b, which demonstrates a peak of RGO@n-SiNWs located at about 22°, contributable to the reduction of GO to RGO [44].

**Fig. 3:**
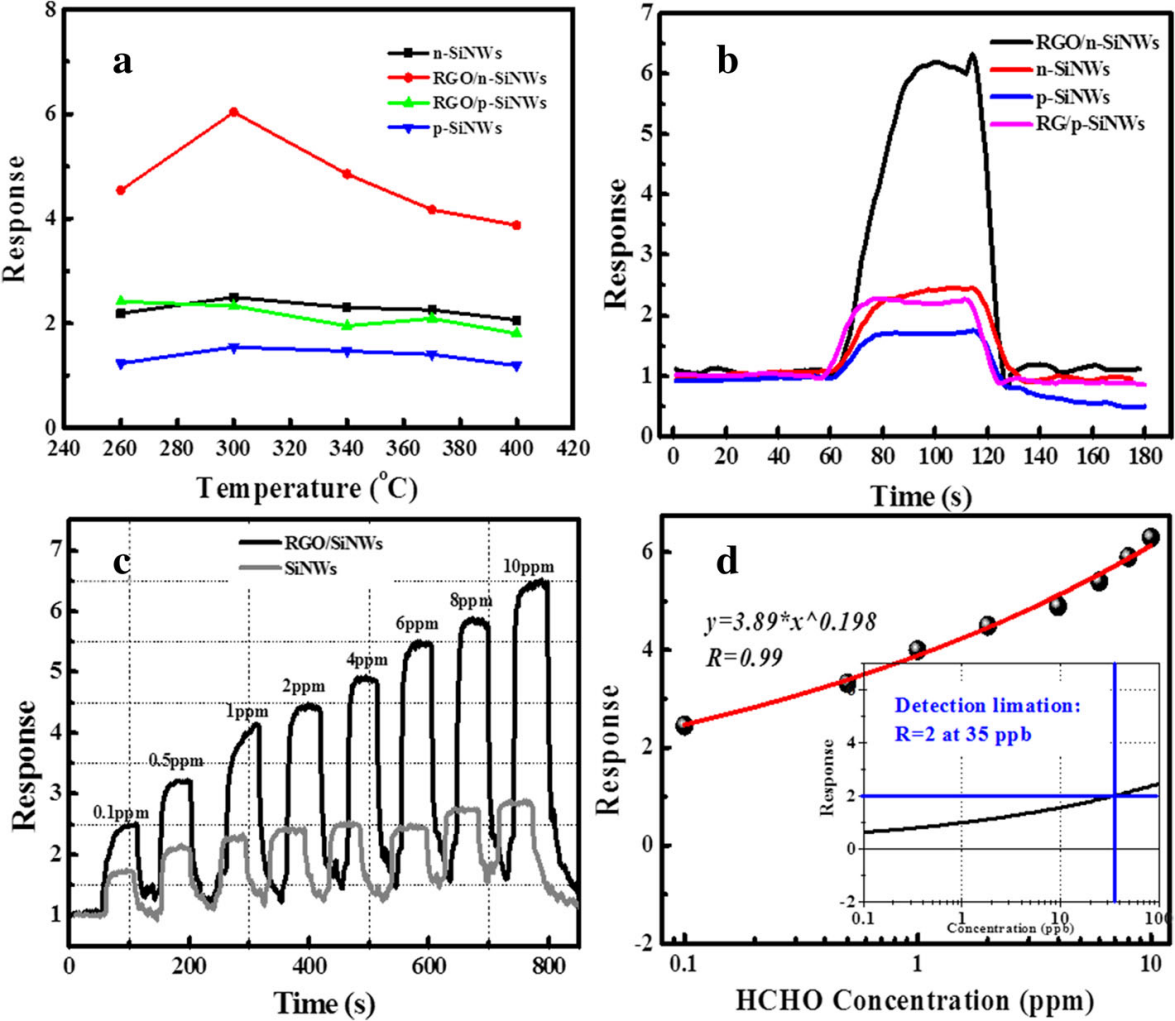
**a** The responses of n-/p-SiNWs, RGO/n- and RGO@p-SiNWs to 10 ppm HCHO at 300 °C. **b** The response of n-SiNWs and RGO@n-SiNWs to 10 ppm HCHO at various temperatures. **c** The dynamic response of n-SiNWs and RGO@n-SiNWs from 0.1 to 10 ppm HCHO. **d** Non-linear fitting of the response of RGO@n-SiNWs at various HCHO concentrations

In an effort to investigate the sensitivity of RGO@SiNWs to HCHO and the optimal device operation temperature, numerous devices based on SiNWs and RGO@ SiNWs were tested at various temperatures. As displayed in Fig. 3a, b, the response of pristine n-SiNWs is higher than that of p-SiNWs. All the devices based on n-SiNWs and RGO@n-SiNWs show the highest response of 2.5 and 6.4 to 10 ppm at 300 °C. In order to evaluate the dynamic response to various gas concentrations based on n-SiNWs and RGO@n-SiNWs in short time, the dynamic test toward HCHO from 0.1 to 10 ppm at 300 °C was performed as displayed in Fig. 3c. It is distinctly observed that the response of n-SiNWs was increased remarkably by wrapping RGO. Meanwhile, the device based on RGO@n-SiNWs has an outstanding response of 2.4 even at a low-concentration of 0.1 ppm, absolutely meeting the criteria of HCHO. As depicted in non-linear fitting in Fig. 3d, the application limitation (*R*_a_/*R*_g_ = 2) was obtained to be 35 ppb, indicating a very low detectable concentration.

**Fig. 4:**
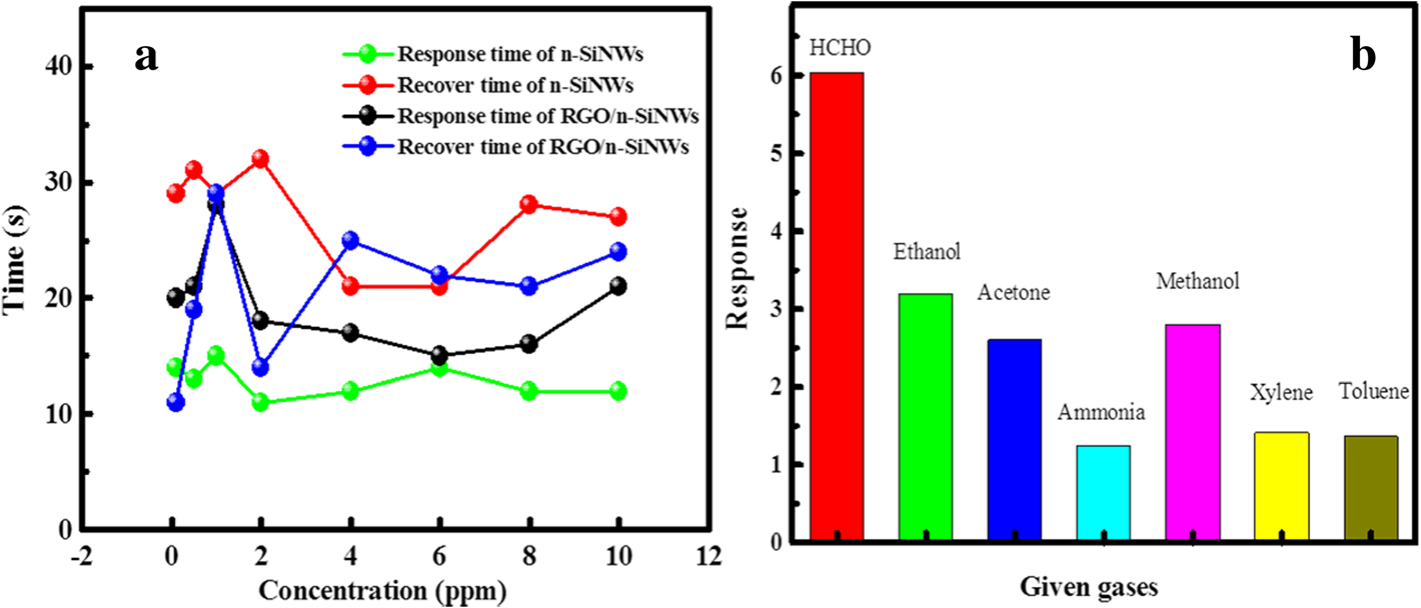
**a** Response and recovery time of n-SiNWs and RGO@n-SiNWs to 0.1 ppm HCHO. **b** The response of n-SiNWs and RGO@n-SiNWs for seven types of common VOCs (10 ppm) at 300 °C

**Fig. 5:**
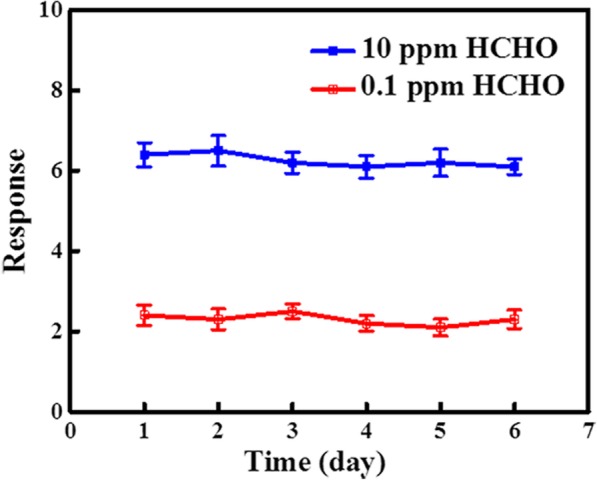
Stability test of n-SiNWs and RGO@n-SiNWs for 0.1 ppm and 10 ppm

Response speed and selectivity are always the important parameters for the practical applications of prepared devices. As indicated in Fig. 4a, both n-SiNWs and RGO@n-SiNWs show extremely short response time (11 and 13 s, respectively), suggesting a relatively fast response. With the purpose to evaluate the selectivity of as-prepared RGO@n-SiNWs sensors, another six typical VOCs (i.e., ethanol, acetone, ammonia, methanol, xylene, and toluene) were employed to examine the sensor selectivity, and the measured results are shown in Fig. 4b, revealing a limited interference to the HCHO detection. The high selectivity to HCHO is resulted by the higher reducibility of HCHO than acetone, ethanol, methanol, toluene, and xylene, as investigated in previous reports [45–47]. Thus, HCHO is more easily oxidized by RGO@n-SiNWs, causing the large decreased resistance. Besides, it is noted that there is almost no response to ammonia for Si sensors [48], because it is not easily oxidized by Si. Apart from selectivity, stability is also a critical challenge in the field of HCHO detection. As investigated in Fig. 5, the response of RGO@n-SiNWs sensors operated at 300 °C changes a little (< 5%) from initial 6.4 to 6.1 after 6 days, indicating an excellent air stability.

**Fig. 6:**
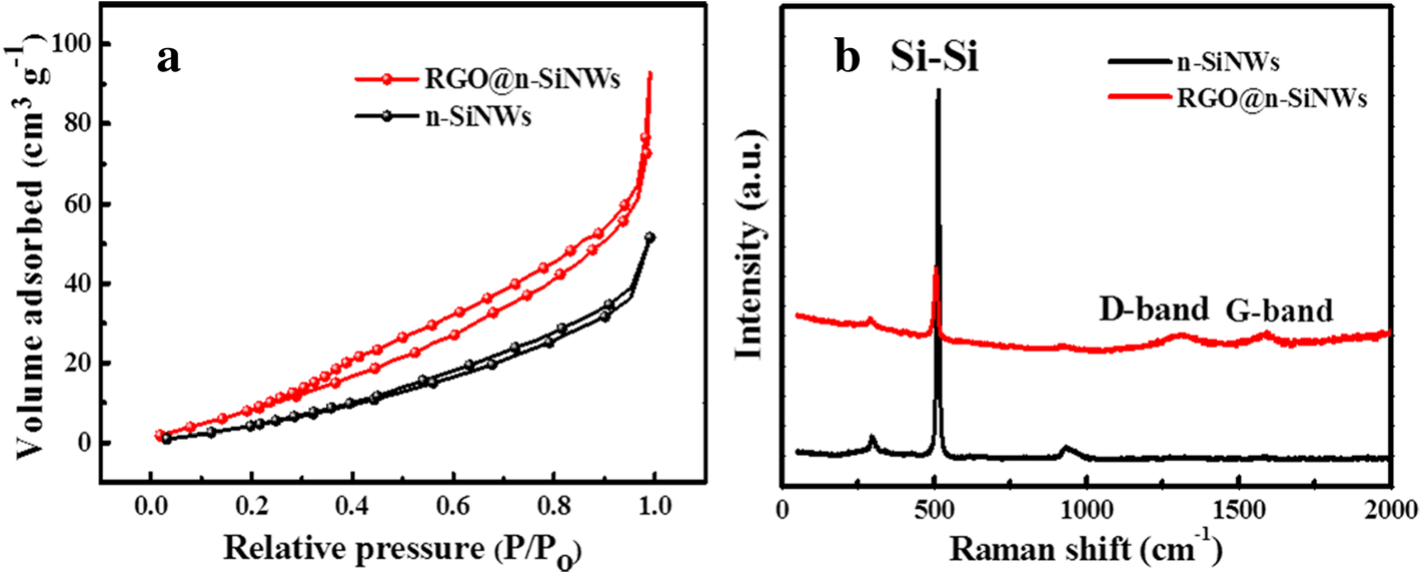
**a** Typical nitrogen adsorption isotherms of n-SiNWs and RGO@n-SiNWs. **b** Raman shift of n-SiNWs and RGO@n-SiNWs, and the zoomed-in Si-Si peaks as shown in inset

The surface-volume ratio (specific surface area) is of great significance to affect gas sensitivity. As studied in nitrogen adsorption-desorption isotherms in Fig. 6a, the surface area is increased from 37.3 m^2^ g^−1^ of n-SiNWs to 74.5 m^2^ g^−1^ of RGO@n-SiNWs, which was originated from the large surface area of RGO. The enlarged specific surface is bound to increase the effective contact-area between targeted gases and samples, thus further improve gas sensitivity. As presented in Raman spectra (Fig. 6b), correlative peaks of Si displayed at 500 and 912 cm^−1^ were observed in RGO@n-SiNWs, demonstrating the presence of Si-Si bonds [49]. Besides, peaks at 1390 and 1590 cm^−1^ are assigned to the D- and G-band peaks of carbon phase due to the disordered and ordered *sp2* bonded carbon, respectively [49], which can infer the presence of reduced graphene oxide. Generally, the *I*_D_/*I*_G_ (the intensity ratio of D and G band) is considered as the most important parameter to evaluate the graphitization degree of carbonaceous materials [49]. The *I*_D_/*I*_G_ is calculated to be 0.72 for RGO@n-SiNWs from Fig. 6b, indicating the high carbonaceous degree of RGO@n-SiNWs.

**Fig. 7:**
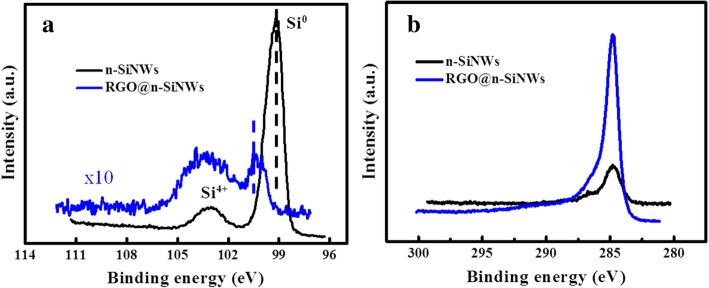
**a** XPS spectra of Si2p peaks for n-SiNWs and RGO@n-SiNWs. **b** XPS spectra of C1s peaks for n-SiNWs and RGO@n-SiNWs

Besides, the chemical compositions of the RGO-SiNWs composites and pristine SiNWs were evaluated by X-ray photoelectron spectroscopy (XPS). As observed in the high-resolution XPS in the vicinity of Si 2p peaks in Fig. 7a, the Si 2p peak intensity of n-SiNWs is decreased distinctly after coating RGO on their surface, while the corresponding C1s peaks intensity of RGO@SiNWs is also enlarged remarkably by comparison with pure SiNWs as observed in Fig. 7b. All of these analyses further prove that the RGO is successfully coated on the surface of SiNWs. Significantly, an evident left shift toward high energy level is revealed in Fig. 7a, resulted by the electron transfer from SiNWs to RGO. XPS data containing the peak position, peak area, surface atomic ratio is demonstrated in Additional file 1: Table S1. The XPS spectra analysis can verify the formation of p-n junction between RGO and SiNWs, which would enhance the transport of electrons generated through the process of HCHO decomposition, and further facilitate the HCHO sensitivity.

**Fig. 8:**
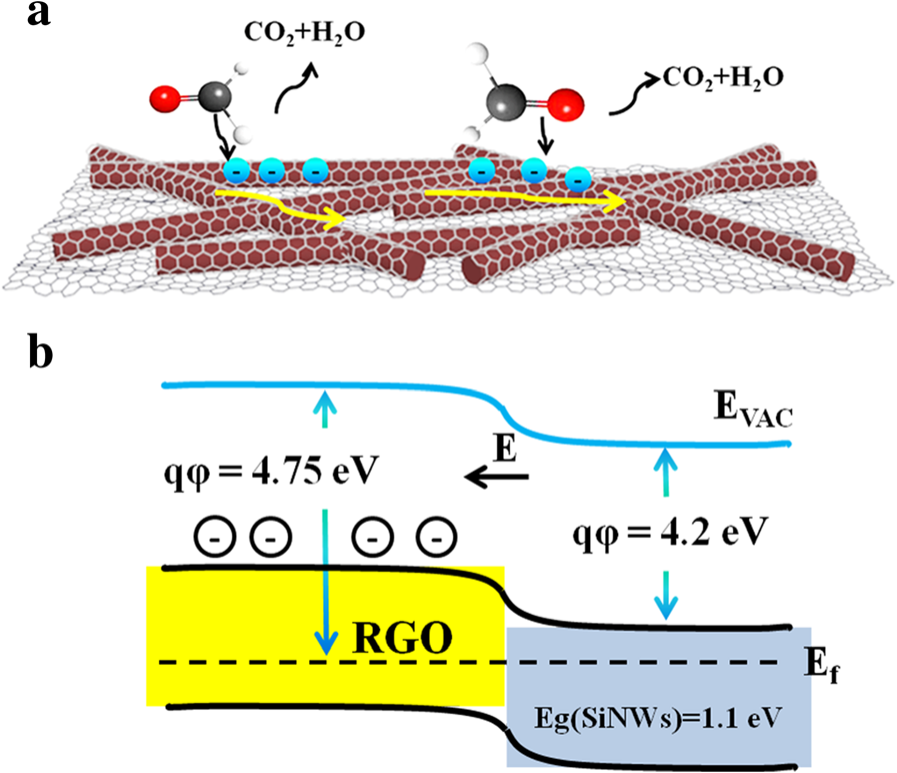
**a** Schematic diagram of the mechanism of HCHO molecules detection. **b** The band structures diagram of the RGO/n-SiNW interface

In an attempt to understand the gas sensing characteristics of RGO@n-SiNWs, the mechanism of the detection toward HCHO is schematically demonstrated. When the as-fabricated sensors were exposed to pure air, the resistance (*R*_a_) will be large due to the chemisorption of oxygen trapping electrons from the material and forming a surface depletion region shown in Eq. (3). While the sensors are exposed to HCHO, the HCHO gas will react with O^−^ and O^2−^, and release electrons to RGO@n-SiNWs, leading to the decrease of resistance (*R*_g_). The reaction process was depicted in Eq. (4) and Fig. 8a.

3 $$ {\mathrm{O}}_2+2{\mathrm{e}}^{-}\to 2{\mathrm{O}}^{-} $$

4 $$ \mathrm{HCHO}\ \left(\mathrm{ads}\right)+2{\mathrm{O}}^{-}\ \left(\mathrm{ads}\right)\to {\mathrm{CO}}_2+{\mathrm{H}}_2\mathrm{O}+2{\mathrm{e}}^{-} $$

Finally, the mechanism of sensitivity enhancement induced by the combination of n-SiNWs and RGO was discussed. The combination of RGO and n-SiNWs can form a p-n junction, as a result of the p-type characterization of RGO with a narrow band gap (0.2 eV~2 eV) [34]. This p-n junction formed between SiNWs and RGO has been reported in many previous reports [50]. To understand how this p-n junction improve the sensitivity, the schematic diagram of band structure is described in Fig. 8b. As illustrated in band structure diagram in Fig. 8b, the electrons are transferred from SiNWs and stored in RGO, forming a depletion layer and a built-in electric field. The electron depletion and built-in voltage would enhance the chemical reaction in Eq. (4) and facilitate the electron transfer, thus enhances the gas sensing performance.

## Conclusions

In summary, SiNWs with high specific surface area are prepared via metal-assisted chemical etching method (MACE), and then are wrapped by reduced graphene oxide (RGO) to form a p-n junction. After wrapping RGO, the specific surface area increases by 1× demonstrated by N_2_ absorption-desorption isotherm. More importantly, due to the formed p-n junction, the RGO@n-SiNWs reveals an outstanding sensitivity and high selectivity toward low concentration HCHO at 300 °C. The response of RGO@n-SiNWs increases about 2× toward 10 ppm HCHO (~ 6.4) at 300 °C than that of pristine n-SiNWs (~ 2.5). The application detection limitation can reach 35 ppb (*R*_a_/*R*_g_ = 2) obtained by non-linear fitting absolutely meeting the safe standard of indoor air. These results provide a promising possibility to precisely detect the low-concentration HCHO, enabling the monitoring the indoor environment.

## Additional file

Additional file 1: **Figure S1.** (a) and (b) Scraping n-SiNWs and p-SiNWs, respectively. Figure S2. (a) TEM images of p-SiNWs. (b) HRTEM image of p-SiNWs together with the corresponding FFT. Table S1. XPS data including the peak position, peak area, surface atomic ratio. (DOC 1938 kb)

## Abbreviations

GO: Graphene oxide
HCHO: Formaldehyde
HRTEM: High-resolution transmission electron microscopy
IAIC: International Agency for Research on Cancer
MACE: Metal-assisted chemical etching
NIOSH: National Institute for Occupational Safety and Health
RGO: Reduced graphene oxide
RGO@n-SiNWs: Reduced graphene oxide-coated n-type silicon nanowires
RGO@SiNWs: Reduced graphene oxide-coated silicon nanowires
SBS: Sick building syndrome
SEM: Scanning electron microscopy
SiNWs: Silicon nanowires
TEM: Transmission electron microscopy
VOCs: Volatile organic compounds
WHO: World Health Organization
XPS: X-ray photoelectron spectroscopy
XRD: X-ray diffraction
