# Supplementary material for: Reduced Graphene Oxide-Coated Si Nanowires for Highly Sensitive and Selective Detection of Indoor Formaldehyde
Source: Nanoscale Res Lett. 2019 Mar 14;14:97. doi: 10.1186/s11671-019-2921-2 (PMC6419648; doi:10.1186/s11671-019-2921-2)
Supplement: Supplementary file 1 — Figure S1. (a) and (b) Scraping n-SiNWs and p-SiNWs, respectively. Figure S2. (a) TEM images of p-SiNWs. (b) HRTEM image of p-SiNWs together with the corresponding FFT. Table S1. XPS data including the peak position, peak area, surface atomic ratio. (DOC 1938 kb) [file 11671_2019_2921_MOESM1_ESM.doc]

**Reduced graphene oxide coated Si nanowires for highly sensitive and selective detection of indoor formaldehyde**

Longfei Song^1,2^**^†^**, Linqu Luo^1^**^†^**, Jianjun Song^1^, Ying Wang^2^, Liping Yang^2,3^, Anqi Wang^2^, Yan Xi^1^, Ning Han^2^,*****, Fengyun Wang^2^,***,** Yunfa Chen^2,3^

^1^College of Physics and State Key Laboratory of Bio-Fibers and Eco-Textiles, Qingdao University, Qingdao 266071, China

^2^State Key Laboratory of Multiphase Complex Systems, Institute of Process Engineering, Chinese Academy of Sciences, Beijing 100190, China

^3^Center for Excellence in Regional Atmospheric Environment, Institute of Urban Environment, Chinese Academy of Sciences, Xiamen 361021, China

Corresponding Authors: ^*^E-mail: [fywang@qdu.edu.cn](mailto:fywang@qdu.edu.cn)**;** ^*^E-mail: [nhan@ipe.ac.cn](mailto:nhan@ipe.ac.cn)


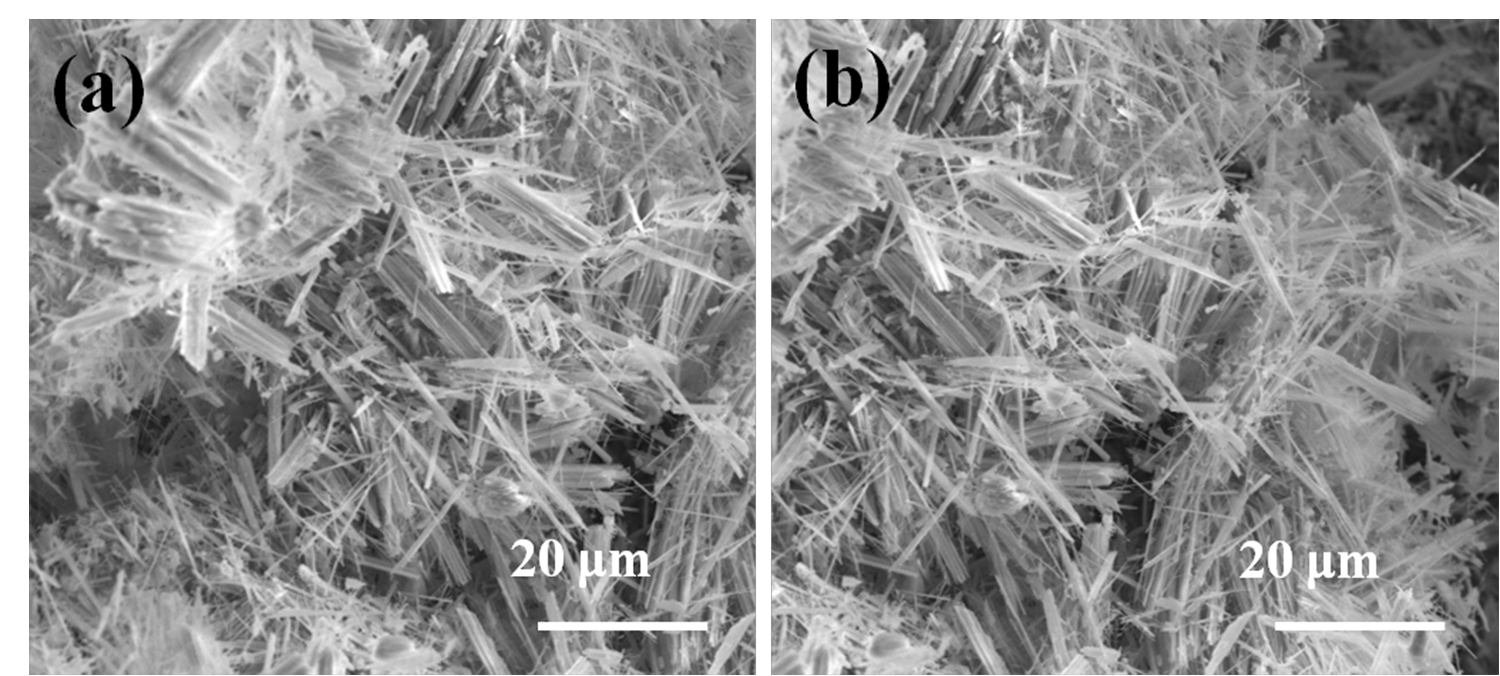


Figure S1. (a) and (b) Scraping n-SiNWs and p-SiNWs, respectively.


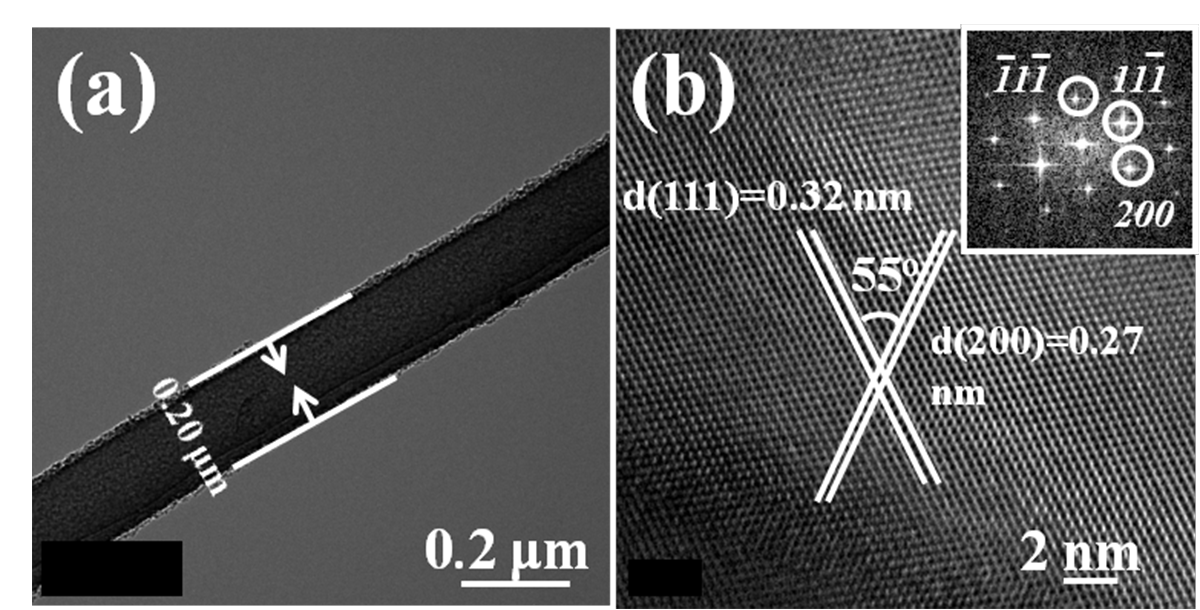


Figure S2. (a) TEM images of p-SiNWs. (b) HRTEM image of p-SiNWs together with the corresponding FFT.

Table S1. XPS data including the peak position, peak area, surface atomic ratio.

| **Samples** | **Si^0^ area** | **Si^4+^ area** | **Si^0^ position** | | **Si^4+^ position** | **C1s area** | **C1s position** | | **Surface atomic ratio** ***** |
| --- | --- | --- | --- | --- | --- | --- | --- | --- | --- |
| **SiNWs** | 33344.2 | 6478.8 | | 99.06 eV | 103.2 eV | 20255.6 | | 284.7 eV | 1.6 |
| **RGO@n-SiNWs** | 869.3 | 100.3 | 100.2 eV | | 103.5 eV | 83763.7 | 284.8 eV | | 0.01 |

*Surface atomic ratio = [S(Si^0^) +S(Si^4+^)]/S(C1s)
